# Evaluation of Eleven Plant Species as Potential Banker Plants to Support Predatory *Orius sauteri* in Tea Plant Systems

**DOI:** 10.3390/insects12020162

**Published:** 2021-02-14

**Authors:** Ruifang Zhang, Dezhong Ji, Qiuqiu Zhang, Linhong Jin

**Affiliations:** State Key Laboratory Breeding Base of Green Pesticide and Agricultural Bioengineering, Key Laboratory of Green Pesticide and Agricultural Bioengineering, Ministry of Education, Guizhou University, Huaxi District, Guiyang 550025, China; gs.rfzhang16@gzu.edu.cn (R.Z.); gs.dzji19@gzu.edu.cn (D.J.); gs.zhangqq18@gzu.edu.cn (Q.Z.)

**Keywords:** *Orius sauteri*, plant-based support system, host plant, tea plant, biological control

## Abstract

**Simple Summary:**

The tea plant is an economically significant beverage crop globally, especially in China. However, tea green leafhoppers and thrips are key pests in Asian tea production systems, causing serious damage to its yield and quality. With growing concerns about pesticide residues on tea and their adverse effects on natural enemies of tea pests, biological pest control is gaining more importance in tea plantations. *Orius sauteri* is a polyphagous predator used as a biological control agent. Here, we reported 11 plants as banker plants to support the predatory *Orius sauteri* in tea plant systems. Among them, white clover, red bean, mung bean, peanut, soybean, kidney bean, bush vetch, smooth vetch, and common vetch were found suitable; red bean performed relatively better than the others.

**Abstract:**

Tea green leafhoppers and thrips are key pests in tea plantations and have widely invaded those of Asian origin. Pesticides are currently a favorable control method but not desirable for frequent use on tea plants. To meet Integrated Pest Management (IPM) demand, biological control with a natural enemy is viewed as the most promising way. *Orius sauteri* are slated to be a natural enemy to tea pests. However, more knowledge of rearing *O. sauteri* and selecting banker plant systems is strongly needed. The reproductive biology evaluation of the egg oviposition and population life parameters of *O. sauteri* under laboratory conditions were examined, and the supporting ability of 11 plant species—motherwort, white clover, red bean, mung bean, peanut, soybean, kidney bean, herba violae, bush vetch, smooth vetch, and common vetch—in a greenhouse was assessed. Most of the selected plants, except for herba violae, performed relatively well with high oviposition quantity and survival. The mean fecundity per female on red bean and motherwort was 148.75 eggs and 148.25 eggs, respectively, and 90.20 eggs for tea plants (the smallest); there also were significant differences. In an experiment to determine the life parameters of *O. sauteri*, all the tested plants, except herba violae, were found to be able to complete the growth and development of the life cycle; there also were significant differences. The intrinsic rate of increase of motherwort and red bean was 1.18 and 1.17, respectively, and higher compared to that of the other plants, including tea plants (1.13). This result of the *O. sauteri* population development index was also confirmed in a greenhouse with the number of motherwort and red beans being as high as 113.33 and 112.67. Since motherwort was found to be susceptible to aphids and powdery mildew in each trial, it cannot be used for intercropping in tea gardens. Among the 11 plants, red bean was found to be the most suitable to support *O. sauteri* in tea plantations.

## 1. Introduction

The tea plant (*Camellia sinensis* (L.) O. Kuntze) is an economically significant beverage crop globally with 5.95 million tons of total tea consumption and around 2.9 million hectares of area harvested [1]. Tea green leafhoppers (*Empoasca vitis Gothe*) and thrips (*Dendrothrips minowai Priesner*) are key pests in Asian tea production systems, including in China [2]. At present, the control of these tea pests is highly dependent on the frequent applications of broad-spectrum insecticides. However, there is a growing concern about pesticide residues in tea, their inconsistent effects on pest populations, and adverse effects on natural enemies of tea pests [3]. At the same time, there is a growing need for sustainable pest management practices and it is necessary to adopt tea pest management practices with a markedly reduced reliance on pesticides [4]; for example, natural enemy-mediated indirect interactions among prey species, the potential for enhancing biocontrol services in agroecosystems [5]. As a result, biological pest control is gaining more importance in tea plantations and selecting a strong breed or utilizing natural enemies can be helpful.

A possible solution for natural enemies is a plant-based support system (banker plant systems) as part of “ecological engineering” [3,4]. This includes nectar plants, habitat plants, trapping plants, indicator plants, shelter plants, etc. [6,7]. Plant-based support systems (banker plant systems) involve the promotion of plant diversity and support the survival and reproduction of natural enemies by providing food, covers for overwintering, and breeding for natural enemies to reduce susceptibility of agricultural crops to native and invasive pests, thus reducing the use of pesticides and environmental pollution [8].

In 1977, Stacey was the first to use plants as a habitat for *Encarsia formosa* and successfully prevented the whitefly (*Trialeurodes vaporariorum*) from damaging greenhouse tomatoes [9]. Since then, creating plant-based support systems that protect natural enemies to achieve long-term effective pest control has become a hot topic in biological control research [10,11]. Waite [12] reported that purple flesh pepper was the most suitable “bank plant” for *Orius insidiosus* to enhance its biocontrol of western flower thrips in greenhouse crops. Zhao [13] used *Calendula officinalis* to expand the population of *Orius sauteri* in greenhouses and improve its control effect on aphids and thrips. Plant resource is a major factor in changing the living environment of predators in the field by adjusting the development of natural enemies, the survival rate of nymphs, and the reproduction rate of adults [7,8,9,10]. Hence, the selectivity of natural enemies for specific host plants plays an important role in biological control [14,15].

*Orius sauteri* (Poppius) *(Heteroptera: Anthocoridae)* is a polyphagous predator used as a natural enemy [16,17,18,19,20]. For example, *O. sauteri* possesses a biological control effect on pest insects in pea pushes [17]; it also has been registered as a biocontrol agent and evaluated with respect to prey selection, reproduction, and predation capability on thrips in eggplant [19]. *O. sauteri* often lays eggs in the petioles, vein tissues, and tender stems of inhabiting plants [21,22]. The life cycle of an *O. sauteri* involves the laying of an egg, the development through nymph of 5 instars, followed by the emergence of a winged adult [21,22]. The eggs are short eggplant-shaped, white, and the egg cover is white and exposed. The nymph in 1st instar-5th instar stage is around 0.54 mm-1.88 mm long. At the end of development, the male larva is basically formed and the female’s oviposition tube begins to differentiate [21,22]. In order to improve the biological control ability of *O. sauteri* against pest thrips and green tea leafhoppers in tea plantations, a plant-based support system is expected to be established. The selection of plants suitable for development of *O. sauteri* populations is fundamental work. In this study, we evaluated the supporting ability of the selected plants, with respect to oviposition or reproductive ability of *O. sauteri* adults, population life parameters, *O. sauteri*’s development from first instar to adult stage, and population growth on different plants beside tea plants as a banker plant.

## 2. Materials and Methods 

### 2.1. Plants

The species and sources of plants involved in this experiment are shown in Table 1. All type of plants in the trials were developed from commercially available seeds with no chemical pretreatment. The seeds were hand sown into seedling trays (48 cm × 15 cm × 8 cm) containing general seedling-raising substrate (pH = 5.5–7.0, organic matter ≥ 20%) in a climate-controlled room (25 ± 1 °C, 70 ± 5% of relative humidity (RH), 16h:8h light (L)/dark (D) at Guizhou University. One month later, seedlings 3-4 cm high were transplanted to plastic pots (diameter 8 cm, height 10 cm). All the plants were manually watered with an N P K fertilizer (20%–20%–20%, N–P–K in mass concentration) solution once a day. Two-year-old tea plants transplanted from the Guizhou tea plantation were cultivated in a climate-controlled room to be used.

### 2.2. Insect Colonies and Food Sources

*O. sauteri* colony-originated adults were obtained from Beijing Kuoye Tianyuan Biotechnology Co., Beijing China. Both nymphs and adults were fed with rice meal moth eggs (*Corcyra cephalonica*) and reared in separate containers in a climate-controlled room (25 ± 1 °C, 70 ± 5 % RH, 16: 8 L/D) at Guizhou University. Frozen-fresh rice meal moth eggs were provided by colleagues at Jilin agriculture University and reproduced in our lab by feeding with wheat bran. The accumulated eggs were made into egg cards with embryo killing treatment and stored in a lab refrigerator (at 5 °C) until they were ready for use. 

Each insect cage (25 cm × 25 cm × 25 cm) was enclosed by a 120-mesh colorless transparent nylon net with a zipper on one side for ventilation and ease to observe and operate. This colony had been maintained without any introduction of “wild” individuals for over three consecutive generations. Green bean pods (*Phaseolus vulgaris* L.) coexisted as an oviposition substrate and also as humidifiers. Every three days, green beans pods were transferred to a new cage to produce discrete even-aged cohorts and egg strips. Cages containing adults lay eggs in green beans pods.

### 2.3. Development of O. sauteri Nymph (Eggs to Adult Stage) in Selected Plant Species

Under a microscope (MDG41, Leica Microsystems), we counted and placed 300 *O. sauteri* eggs laid on the same day in each of the 12 cages (25 cm × 25 cm × 25 cm) with 12 tested plants, in a climate-controlled room (25 ± 1 °C, 70 ± 5% RH, 16: 8 L/D). Fresh rice meal moth eggs were added to feed the *O. sauteri* and the test plants were watered every day. Hatching eggs, development time of *O. sauteri* nymph (from 1st instar to adult stage), number of nymph and adults, and number of females were recorded daily, and then emergence rate was calculated. 

During the observation process, the plants with newly laid eggs were replaced by new plants every three days until all the adults were dead. The experiment with three parallel cages for each plant was repeated thrice.

### 2.4. Evaluation on Reproductive Capacity of O. sauteri Adults on Different Host Plants 

Adults taken from the rearing colony were sorted according to male and female by observing their abdomen under a microscope (MDG41, Leica Microsystems, Heerbrugg, Switzerland)—the male and female reproductive organs are different (symmetrical for females and asymmetrical for males) [23]. In order to not miss the oviposition period, a newly emerged pair of adults (1 ± 1 day) were placed in a colorless transparent plastic tetrapod (189 cm × 93 cm × 65 cm) containing a test plant. Sufficient fresh moth egg cards were provided for feeding *O. sauteri*. If the male was found dead during the experiment, another male was supplemented to make sure the female mated. In order to accurately count the number of newly laid eggs, the host plants with laid eggs were replaced by the same fresh plant every day. The *O. sauteri* eggs attached to the exchanged host plant was counted and then maintained in another insect cage. All the new eggs were recorded until adult death. Twelve plant species, including tea plants, was tested in three parallel cages and each trial was repeated six times.

### 2.5. O. sauteri Growth in Tea Plants Intercropped with 11 Tested Plants

The plants were evaluated for their suitability to support *O. sauteri* by intercropping in a greenhouse field at Guizhou University. Tea plants intercropped with 11 test plants were set in a 50 cm × 25 cm × 25 cm cage. 3♂ and 3♀*O. sauteri* adults (1 ± 1 days) were released into the cage with sufficient rice moth eggs as food. On the seventh day, the data was counted and 3♂ and 3♀*O. sauteri* adults (1 ± 1 days) were released again, and then the number of adults and nymphs of *O. sauteri* was recorded every two weeks, continuing thus for seven weeks. To record their number accurately, *O. sauteri* were brushed off the plant, counted, and then released back into the cage for continued development. The plants were regularly watered. The experiment with three parallel cages for each plant was repeated thrice.

### 2.6. Data Analysis and Calculations

Hatching rate = number of hatching nymphs/ number of eggs; 

Emergence rate = number of emerging adults/number of 3-4 instar nymphs;

The life table construction parameters were carried out according to the following formula [20,21,22]:

Population trend index = number of expected next generation eggs/ number of previous generation eggs;

Expected oviposition amount of the next generation = standard oviposition amount × normal female adult;

The standard oviposition amount was the maximum oviposition amount of a female observed in the actual experiment;

Generation survival rate = the adult Lx/the initial egg size of that generation;

Net reproductive rate (*R*_0_) = ∑L_x_ M_x_;

Mean generation time (T) = ∑L_x_ M_x_ X/∑L_x_ M_x_

Intrinsic rate of increase (r_m_) = ln *R*_0_/T;

Finite increase rate (λ) = exp (r_m_);

Population doubling time (d) = ln2/r_m_; 

x represents the interval between two observations and a three-day break was set here;

L_x_ is the survival rate at the x day period; 

M_x_ is the average number of female offspring per female adult during x period;

X represents the developmental stage (egg-nymph-adult);

Lx is the number of surviving insects at the beginning of the X period.

*O. sauteri* insect development in every test was investigated and recorded, including fecundity per female (egg number per female), oviposition period, number of new-hatching nymphs, nymph development time (mean time from hatching to adult eclosion), emergence rate. One-way ANOVA in SPSS was used to analyze those data. The means were compared by Tukey’s test at *p* < 0.05 level; a Type I error rate (a) of 0.05 was used to test for significance. 

## 3. Results

### 3.1. Development of Orius sauteri Nymph (1st Instar to Adult)

Each generation of *O. sauteri* was composed of an egg, nymph (1st instar-5th instar) and adult. We carefully recorded the hatching rate (percentage of the eggs hatched), nymph development time (from 1st instar to adult) and emergence rate. It was showed that hatching rate (F = 1.115; df = 11, 24; *p* = 0.392) (Table 2) in each host plant was at a similar level, and all matched that of the tea plants. Among them, white clover, motherwort, and herba violae supported a higher hatching rate with 93.33%, 91.37%, and 90.30% for *O. sauteri.* For nymph development time (F = 12.681; df = 11, 24; *p* < 0.001) (Table 2) from 1st instar to adult, a close level was exhibited in the tested plants excepted for herba violae and the difference among them was less than two days. However, the *O. sauteri* nymph development time in herba violae was as low as around 1.50 days and the nymphs were found dead at around the 1st instar. With regard to emergence rate (F= 22.841; df = 11, 24; *p* < 0.001) (Table 2), they were significantly diverse. Data of the emergence rate (42.35%-76.67%) in different plant species except herba violae were superior than that of the tea plants (33.675%), where the red bean (76.67%), motherwort (74.93%), and mung bean (71.26%) hold a relatively higher rate, but there were no significant differences between them. Due to the short survival time (1.5 day), the *O. sauteri* nymph development was found to be stagnant; no emergence appeared in the herba violae plant. Therefore, 11 tested plants, except herba violae, possess a better suitability with significant differences compared to the tea plants.

### 3.2. Reproductive Capacity of Orius sauteri Adults 

One pair of newly emerged adults (1 ± 1day) were placed and reared in different cages (189 cm × 93 cm × 65 cm) containing a different test plant. The number of eggs laid by each female was used as the principal physiological standard of fecundity (eggs per female) reproductive capacity of *O. sauteri* adults. The plant species did have an significant effect on single-female total fecundity (F = 5.828 df = 11, 38; *p* < 0.001), oviposition period (F = 5.818; df = 11, 38; *p* < 0.001) (Table 3); taking tea plants (average 90.20 eggs, 12.00 days) (Table 3) as a reference, the data of the single-female total fecundity and oviposition period differed greatly, for 10 plant species, except herba violae, which were larger, with red bean (mean 148.75 eggs, 20.50 days) and motherwort (mean 148.25 eggs, 19.25 days) (Table 3) being significant, and herba violae not being significant compared to tea plants. The tested plant species had a significant effect on the reproductive capacity of *O. sauteri*.

### 3.3. Population Life Parameters of Orius sauteri on Different Host Plants

**Figure 1:**
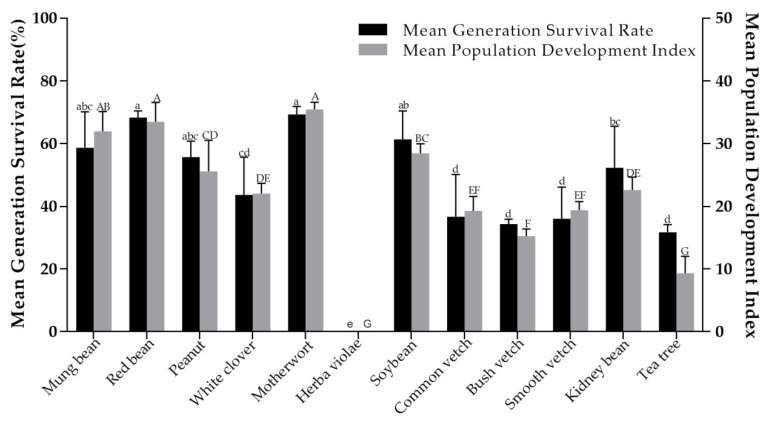
Mean generation survival rate and population development index of *Orius sauteri* (whole life) reared on 11 plant species (tea plant as the reference plant) in a growth chamber at 25 ± 1 °C, 70 ± 5 % RH, 16h: 8 h L/D. The means followed by different letters are significantly different from each other (Tukey’s test, *p* = 0.05).

The different stages (egg, nymph and adult) of generation development were recorded and the life parameters of *O. sauteri* were calculated and evaluated, according to the method [24,25,26] described in Section 2.6. There were significant differences in net reproductive rate (*R*_0_) (F = 29.375; df = 11, 22; *p* < 0.001) (Table 4), mean generation time (T) (F = 24.140; df = 11, 22; *p* < 0.001) (Table 4), intrinsic rate of increase (r_m_) (F = 12.204; df = 11, 22; *p* < 0.001) (Table 4), finite increase rate (λ) (F = 12.291; df = 11, 22; *p* < 0.001) (Table 4), population doubling time (F = 11.118; df = 11, 22; *p* < 0.001) (Table 4), generation survival rate (F= 15.983; df = 11, 24; *p* < 0.001) (Figure 1), and population development index (F= 54.486; df = 11, 24; *p* < 0.001) (Figure 1). To evaluate significant differences, the life parameters of *O. sauteri* on different host plants were comprehensively analyzed. Compared to tea plants, the other 10 plants showed a relatively better index value with finite increase rate (λ), intrinsic rate of increase (r_m_), net reproductive rate (R_0_) generation survival rate, and population development index. There was no significant difference between the net reproductive rate of red bean (76.74%), motherwort (72.89%), and mung bean (65.01%) though they were obviously higher than those of the other eight plants. With regards to population doubling time, tea plants showed the longest (5.19 d) and motherwort the shortest (3.90 d), with a significant difference. For population doubling time, the longer the population doubling time, the more unfavorable was the population development of *O. sauteri*. It is easy to see that, for herba violae, the nymphs in that treatment did not survive to complete the life development with zero generation survival. Thus, herba violae is obviously not suitable to be used as a host plant for *O. sauteri*.

### 3.4. O. sauteri Growth in Tea Plants Intercropped with 11 Tested Plants

Based on the growth capacity of *O. sauteri* population evaluation, the suitability of plant species as host plants was evaluated by intercropping with tea plants in a greenhouse field.

**Figure 2:**
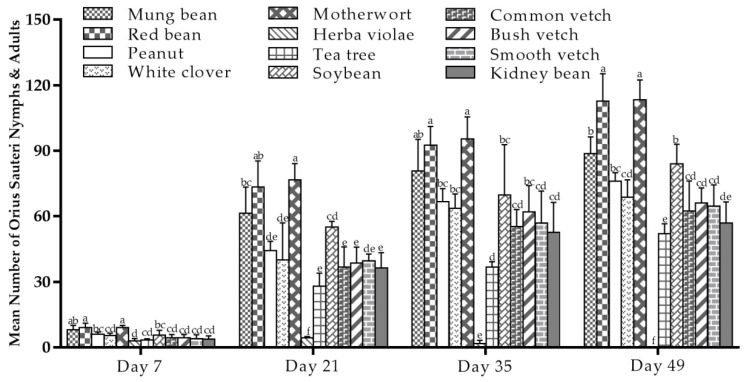
Mean number (±SE) of *O. sauteri* nymphs and adults reared on 11 different host plants (tea plants as the reference) in a greenhouse in a climate-controlled room at Guizhou University, China, recording from 14 December 2018 (day 7) to 31 January 2019 (day 49). The means followed by different letters are significantly different from each other (Tukey’s test, *p* = 0.05).

As shown in Figure 2, there were significant differences in the populations of *O. sauteri* developing on different plant species (F = 6.862; df = 11, 26; *p* < 0.001 (Day 7); F= 16.197; df = 11, 26; *p* < 0.001 (Day 35); F =15.006; df = 11, 26; *p* < 0.001 (Day 21) and F = 38.380; df = 11, 26; *p* < 0.001 (Day 49)). Compared to the tea plants, the number of nymphs and adults was higher on 10 other plant species, except for herba violae. Overall, the population of *O. sauteri* on each species increased over seven weeks in greenhouse conditions and increased steadily with a significantly higher number on red bean and motherwort than on the other plant species (Figure 2). Herba violae had extremely lower populations of *O. sauteri* throughout the recording period, eventually disappearing completely.

## 4. Discussion

The population development of *O. sauteri* on plants is a necessary prerequisite for establishing a plant-based support system in tea plantations. The life table is an important method to study insect population dynamics, evaluating various pest control measures, formulating quantitative forecasting models, and implementing scientific pest control [27,28,29]. In this experiment, in the category of intrinsic rate of increase of 10 plant species, except herba violae, the minimum was 0.13 (Table 4) for tea plants and the maximum was 0.18 for motherwort (Table 4), which was a significant difference. This shows that the population development of *O. sauteri* was different in different host plant environments. When motherwort was used as the host plant, the population of the next generation of *O. sauteri* had 35.4 times increase in previous generation (the largest compared with other plants, and the red bean second had 33.47 times increase; the smallest was for the tea plants—9.34 times) (Figure 1). Generation survival rate of the population was motherwort 69.33%, max; tea plants 31.67%, min (Figure 1). These data are sufficient to show that 10 plant species, except *Herba violae,* can provide supporting conditions for cage bioassay in the development of *O. sauteri*.

Evaluating the plant species for their ability to support *O. sauteri* population growth over multiple generations in a seven-week period provided practical information on the potential to establish plant-based support systems in tea plantation. When compared to the tea plants, the number of *O. sauteri* increased and the population in the 10 plants showed a consistently increasing trend when intercropped with tea plants in a greenhouse. In particular, motherwort (mean 113.33, seventh week) and red bean (mean 112.67, seventh week) (Figure 2) showed high ability, compared to the other plant species, and were expected to be suitable to be intercropped with tea plants to establish plant-based support systems. 

It should be mentioned that the motherwort might fall into disuse because it was found to susceptible to aphids and powdery mildew in greenhouse conditions. The susceptibility to pests and diseases should be considered as a factor in the selection of appropriate candidate supporting plant species [30]. Another exception is that no *O. sauteri* nymphs emerged into adults on herba violae in this study. Almost all the newly hatched nymphs died before the second instar. The finding that utilization of herba violae has a negatively effect on growth parameters in consistent with previous studies [31,32].

There are also other factors affecting the development of *O. sauteri* on the host plants. For example, studies have shown that the oviposition quantity of *O. sauteri* was higher in nitrogen-rich plant systems over nitrogen-poor ones [33,34]. The selected plant in the present study, except herba violae and motherwort, is the *Leguminosae* (*Fabeceae*) family with potential high nitrogen content and they consistently performed relatively well. Although motherwort is not a legume plant, its effect is also very good; thus, further research is needed.

As discovered in the present study, white clover, red bean, mung bean, peanut, soybean, kidney bean, bush vetch, smooth vetch, common vetch, and red bean supported constant populations of *O. sauteri* in greenhouse conditions and could be recommended for intercropping in tea plantation to establish plant-based support systems. The effect of red bean was found to be slightly better than the others.

This experiment was only carried out in experimental conditions and in a greenhouse, not in a field. Studies have found that there may be differences between experimental data and field data [35]. Therefore, future studies are required to determine the dispersal of *O. sauteri* through greenhouse and field tests as well as the determining effects on their searching behavior when prey is scarce.

## 5. Conclusions

In this research, we studied 11 plants as banker plants to support predatory *O. sauteri* in tea plantation systems. Most of the selected plants, except for herba violae, performed relatively well with high oviposition quantity and survival. Since motherwort is susceptible to aphids and powdery mildew, it cannot be a candidate for intercropping in tea gardens. Therefore, it could be concluded that, among the 11 plants studied, white clover, red bean, mung bean, peanut, soybean, kidney bean, bush vetch, smooth vetch, and common vetch were found suitable to support *O. sauteri*. Of these, red bean had superior performance.

## Figures and Tables

**Table 1:** Plant species and source access.

No.	Common Name	Latin Name/Family	Source *
1	Motherwort	*Leonurus artemisia (Lour.) S. Y. Hu/Labiatae*	Blue Sky Seed Industry
2	White clover	*Trifolium repens./Leguminosae*	Blue Sky Seed Industry
3	Bush vetch	*Vicia villosa Roth./Leguminosae*	Blue Sky Seed Industry
4	Common vetch	*Vicia sativa L./Leguminosae*	Blue Sky Seed Industry
5	Smooth vetch	*Vicia glabrescens Koch./Leguminosa* *e*	Blue Sky Seed Industry
6	Herba violae	*Viola philippica./Violaceae*	Blue Sky Seed Industry
7	Soybean	*Glycine javanica L./Leguminosae*	Guizhou Dasheng Seed Industry Co., Ltd.
8	Mung bean	*Vigna radiata (Linn.) Wilczek/Leguminosae*	Guizhou Dasheng Seed Industry Co., Ltd.
9	Red bean	*Vigna angularis (Willd.) Ohwiet Ohashi/Leguminosae*	Guizhou Dasheng Seed Industry Co., Ltd.
10	Peanut	*Arachis hypogaea Linn./Leguminosae*	Guizhou Dasheng Seed Industry Co., Ltd.
11	Kidney bean	*Phaseolus vulgaris Linn./Leguminosae*	Dezhou De Vegetable Seed Industry Co., Ltd.
12	Tea plant	*Camellia sinensis (L.) O. Ktze*/*Theaceae*	Guizhou Tea plantation

* All source locations are within China.

**Table 2:** Development parameters of *O. sauteri* nymph on 11 plant species.

Test Plants	Hatching Rate (%)	Nymph Development Time (d)	Emergence Rate (%)
Mung bean	87.62 ± 3.06 a	13.00 ± 0.58 a	71.26 ± 1.90 a
Red bean	87.62 ± 3.06 a	12.33 ± 0.88 a	76.67 ± 3.16 a
Peanut	89.12 ± 1.82 a	11.00 ± 1.45 a	54.55 ± 3.54 bc
White clover	93.33 ± 0.66 a	14.00 ± 0.58 a	54.69 ± 6.52 bc
Motherwort	91.37 ± 2.39 a	12.33 ± 0.88 a	74.93 ± 3.35 a
Herba violae	90.30 ± 2.36 a	1.50 ± 0.50 b	0.00 ± 0.00 f
Soybean	86.44 ± 2.23 a	12.33 ± 0.88 a	67.24 ± 3.82 ab
Common vetch	86.40 ± 2.36 a	12.33 ± 1.45 a	42.35 ± 4.27 cd
Bush vetch	85.72 ± 2.15 a	12.00 ± 1.15 a	45.40 ± 6.57 cd
Smooth vetch	86.41 ± 2.00 a	12.33 ± 0.33 a	51.94 ± 3.07 c
Kidney bean	88.34 ± 2.64 a	12.00 ± 1.73 a	56.56 ± 7.70 bc
* Tea plant	86.50 ± 2.13 a	13.80 ± 0.42 a	32.51 ± 3.71 d

The means followed by different letters are significantly different from each other (Tukey’s test, *p* = 0.05). * Tea plants served as the reference plant at 25 ± 1 °C, 70 ± 5 % RH, 16h: 8 h L/ D.

**Table 3:** Reproductive capacity of *O. sauteri* adults.

Test Plants	Fecundity (Eggs per Female)	Oviposition Period (Day)
Mung bean	124.75 ± 12.99 abc	15.25 ± 1.31 abcd
Red bean	148.75 ± 8.06 a	20.50 ± 1.55 a
Peanut	121.00 ± 9.46 abc	16.75 ± 1.38 abcd
White clover	132.25 ± 10.19 ab	18.00 ± 1.22 abc
Motherwort	148.25 ± 11.02 a	19.25 ± 1.93 ab
Herba violae	94.60 ± 5.40 bc	11.60 ± 0.75 d
Soybean	122.25 ± 5.82 abc	16.00 ± 1.08 abcd
Common vetch	103.50 ± 4.63 bc	14.75 ± 0.85 bcd
Bush vetch	119.75 ± 8.41 abc	14.75 ± 1.03 bcd
Smooth vetch	100.00 ± 6.72 bc	13.25 ± 0.85 cd
Kidney bean	117.75 ± 6.80 abc	14.75 ± 0.85 bcd
* Tea plants	90.20 ± 4.85 c	12.00 ± 0.71 d

The means followed by different letters are significantly different from each other (Tukey’s test, *p* = 0.05). * Tea plants served as the reference plant at 25 ± 1 °C, 70 ± 5 % RH, 16h: 8 h L/D.

**Table 4:** The population life parameters of *O. sauteri* (whole life) reared on 11 plant species at 25 ± 1 °C, 70 ± 5 % RH, 16h: 8 h L/ D.

Test Plants	Net Reproductive Rate (R_0_)	Mean Generation Time (T)/d	Intrinsic Rate of Increase (r_m_)	Finite Increase Rate (λ)	Population Doubling Time (t)/d
Mung bean	65.01 bc	26.87 a	0.15 cd	1.17 cd	4.47 bc
Red bean	76.75 a	26.26 ab	0.16 abc	1.18 abc	4.20 c
Peanut	44.58 de	22.03 f	0.17 ab	1.19 ab	4.02 d
White clover	54.44 cd	25.90 bc	0.15 cd	1.17 cd	4.49 bc
Motherwort	72.89 ab	24.08 e	0.18 a	1.19 a	3.90 d
Herba violae	/	/	/	/	/
Soybean	64.96 bc	25.41 bc	0.16 bc	1.18 bc	4.23 c
Common vetch	40.38 e	23.70 e	0.16 cd	1.17 cd	4.45 de
Bush vetch	39.73 ef	25.15 cd	0.15 de	1.16 de	4.74 b
Smooth vetch	38.46 ef	25.15 cd	0.14 de	1.16 de	4.79 b
Kidney bean	35.86d ef	24.44 de	0.15 de	1.16 de	4.75b
* Tea plants	29.35 f	25.21 cd	0.13 e	1.14 e	5.19 a

The means followed by different letters are significantly different from each other (Tukey’s test, *p* = 0.05). * Tea plants served as the reference plant.

## Data Availability

The data presented in this study are available from the corresponding author with reasonable request

## References

[1] Singh H.R., Hazarika P. (2020). Biotechnological Approaches for Tea Improvement in Biotechnological Progress and Beverage Consumption.

[2] Zhou Z.Y., Hu B.J., Xu L., Hu F., Li C.G., Gao T.C., Su W.H. (2016). Selection of high-efficient safe pesticides for controlling tea lesser leafhopper (Empoasca vitis). Agric. Sci. Technol..

[3] Settele J., Biesmeijer J., Bommarco R. (2008). Switch to ecological engineering would aid independence. Nat. Cell Biol..

[4] Gurr G.M., Wratten S.D., Snyder W.E., Read D.M.Y. (2012). Biodiversity and Insect Pests: Key Issues for Sustainable Management.

[5] Chailleux A., Mohl E.K., Alves M.T., Messelink G.J., Desneux N. (2014). Natural enemy-mediated indirect interactions among prey species: Potential for enhancing biocontrol services in agroecosystems. Pest Manag. Sci..

[6] Frank S.D. (2010). Biological control of arthropod pests using banker plant systems: Past progress and future directions. Biol. Control..

[7] Chen X.X., Liu Y.Q., Ren S.X., Zhang F., Zhang W.Q., Ge F. (2014). Plant-mediated support system for natural enemies of insect pests. Chin. J. Appl. Entomol..

[8] Jaworski C.C., Chailleux A., Bearez P., Desneux N. (2015). Predator-mediated apparent competition between pests fails to prevent yield loss despite actual pest population decrease. J. Pest Sci..

[9] Stacey D.L. (1977). ’Banker’ Plant Production of Encarsia formosa Gahan and its Use in the Control of Glasshouse Whitefly on Tomatoes. Plant Pathol..

[10] Collier T., Steenwyk R.V. (2004). A critical evaluation of augmentative biological control. Biol. Control..

[11] Lu Y., Wu K., Jiang Y., Guo Y., Desneux N. (2012). Widespread adoption of Bt cotton and insecticide decrease promotes biocontrol services. Nat. Cell Biol..

[12] Waite M.O., Scott-Dupree C.D., Brownbridge M., Buitenhuis R., Murphy G. (2013). Evaluation of seven plant species/cultivars for their suitability as banker plants for Orius insidiosus (Say). BioControl.

[13] Zhao J., Guo X., Tan X. (2017). Using Calendula officinalis as a floral resource to enhance aphid and thrips suppression by the flower bug Orius sauteri (Hemiptera: Anthocoridae). Pest Manag. Sci..

[14] Wratten S.D., Gillespie M., Decourtye A., Mader E., Desneux N. (2012). Pollinator habitat enhancement: Benefits to other ecosystem services. Agric. Ecosyst. Environ..

[15] Wäckers F.L., Van Rijn P.C.J. (2012). Pick and Mix: Selecting Flowering Plants to Meet the Requirements of Target Biological Control Insects. Biodiversity and Insect Pests.

[16] Landis D.A., Wratten S.D., Gurr G.M. (2000). Habitat Management to Conserve Natural Enemies of Arthropod Pests in Agriculture. Annu. Rev. Èntomol..

[17] Li H.L., Li P., Zhang H., Wang D.F., Li L.D., Zeng M.S., Wu G.Y., Wang Q.S. (2019). Predation of Orius sauterion pest insects of pea pushes. Acta Teas Sin..

[18] Tan X.L., Wang S., Liu T.X. (2014). Acceptance and suitability of four plant substrates for rearing Orius sauteri (Hemip.tera: An-thocoridae). Biocontrol Sci. Techn..

[19] Nagai K., Yano E. (2000). Predation by Orius sauteri (Poppius) (Heteroptera: Anthocoridae) on Thrips palmi Karny (Thysanoptera: Thripidae). Functional response and selective predation. Appl. Èntomol. Zool..

[20] Yin J., Gao X.G., Wu Y.Q. (2013). Thrips control on the greenhouse eggplant by releasing Orius sauteri (Heteroptera: Anthocoridae). Chin. J. Biol. Control.

[21] Kiman. Z.B., Yearga. K.V. (1985). Development and reproduction of the predator Orius insidiosus (Hemiptera: Anthocoridae) reared on diets of selected plant material and arthropod prey. Ann Entomol Soc Am..

[22] Yoshitaka N., Yoshimi H. (1999). Effects of Prey Availability on Longevity, Prey Consumption, and Egg Production of the Insect Predators Orius sauteri and O. tantillus (Hemiptera: Anthocoridae). Ann. Entomol. Soc. Am..

[23] Slater J.A., Triplehorn C.A., Johnson N.F. (2005). Order hemiptera: True bugs, cicadas, hoppers, psyllids, whiteflies, aphids, and scale insects. Borror and Delong’s Introduction to the Study of Insects.

[24] Fan J.S., Zhang L.X., Wang G.Q., Ma X., Han X. (2016). Life tables for experimental populations of Loxostege sticticalis (Lepidoptera: Crambidae) on five host plants. Plant Prot..

[25] Busvine J.R. (1980). Revised method for spider mites and their eggs (e.g., Tetranychus sp. and Panonychus ulmi Koch). FAO method No. 10a. Recommended Methods for Measurement of Pest Resistance to Pesticides.

[26] Shih C.-I.T., Poe S.L., Cromroy H.L. (1976). Biology, Life Table, and Intrinsic Rate of Increase of Tetranychus urticae1. Ann. Èntomol. Soc. Am..

[27] Ding Y.Q. (1980). Population Mathematical Principles and Application of Insect Ecology.

[28] Gao S.K., Yang Z.Q. (2015). Application of life table in pest biological control. Chin. J. Biol. Control.

[29] Sun R.Y. (2001). The Principle of Animal Ecology.

[30] Wong S.K., Frank S.D. (2013). Pollen increases fitness and abundance of Orius insidiosus Say (Heteroptera: Anthocoridae) on banker plants. Biol. Control.

[31] Wu H., Zhan Y., Chen H.F., Li J., Jian H., Zhang W.G., Feng Y.L., Yang S.L. (2016). Rapid identification of chemical constituents in violayedoensis by UHPLC-ESI-Q-TOF-MS/MS. China J. Exp. Tradit. Med. Formulae.

[32] Oshima N., Narukawa Y., Takeda T., Kiuchi F. (2012). Collagenase inhibitors from Viola yedoensis. J. Nat. Med..

[33] Groenteman R., Guershon M., Coll M. (2006). Effects of leaf nitrogen content on oviposition site selection, offspring performance, and intraspecific interactions in an omnivorous bug. Ecol. Èntomol..

[34] Liman A.-S., Dalin P., Björkman C. (2017). Enhanced leaf nitrogen status stabilizes omnivore population density. Oecologia.

[35] Wong S.K., Frank S.D. (2012). Influence of banker plants and spiders on biological control by Orius insidiosus (Heteroptera: Anthocoridae). Biol. Control.

